# Amyloid beta-42 Levels in Companion Dog Brains Correlate with Age and Cognitive Function

**DOI:** 10.1093/geroni/igaa057.3273

**Published:** 2020-12-16

**Authors:** Silvan Urfer, Martin Darvas, Dirk Keene, Kálman Czeibert, Enikő Kubinyi, Sára Sándor, Franco Guscetti, Matt Kaeberlein

**Affiliations:** 1 University of Washington, Seattle, Washington, United States; 2 Etövös Loránd University, Budapest, Budapest, Hungary; 3 University of Zurich, Zurich, Zurich, Switzerland

## Abstract

The privately owned companion dog is an increasingly important model in aging research because it shares the human environment, is exposed to similar environmental risk factors, receives comparable medical care, and develops many of the same age-related pathologies. One such pathology is Canine Cognitive Dysfunction (CCD), which shares many of the clinical features of human Alzheimer’s Disease (AD), including progressive loss of cognitive function, loss of normal sleep patterns, increased anxiety, and aimless wandering. Amyloid-beta 42 (Aβ42) plaques similar to these found in humans with AD are known to naturally occur in the brains of aged dogs, making them an intriguing potential model for AD in humans. As part of the Dog Aging Project (www.dogagingproject.org), we studied frozen samples taken from the frontal cortex, medial temporal cortex, entorhinal cortex, and hippocampus of n=24 companion dogs of various ages that were euthanized for unrelated health reasons and donated by their owners. Brains were removed and frozen within 4 hours post mortem. Using a novel quantitative Luminex assay, we found a significant correlation between age and Aβ42 levels in all of these brain regions, as well as a significant correlation between Aβ42 levels and cognitive function scores as measured by the Canine Cognitive Dysfunction Scale. We will now investigate histopathology in the same dogs and brain regions, and investigate whether we can also measure Tau and pTau in these samples using Luminex and mass spectrometry.

